# Association between *MIF* gene promoter rs755622 and susceptibility to coronary artery disease and inflammatory cytokines in the Chinese Han population

**DOI:** 10.1038/s41598-021-87580-6

**Published:** 2021-04-13

**Authors:** Jun-Yi Luo, Bin-Bin Fang, Guo-Li Du, Fen Liu, Yan-Hong Li, Ting Tian, Xiao-Mei Li, Xiao-Ming Gao, Yi-Ning Yang

**Affiliations:** 1 State Key Laboratory of Pathogenesis, Prevention and Treatment of High Incidence Diseases in Central Asia, Department of Cardiology, First Affiliated Hospital of Xinjiang Medical University, 137 Liyushan South Road, Urumqi, 830054 China; 2 Xinjiang Key Laboratory of Cardiovascular Disease Research, Clinical Medical Research Institute of Xinjiang Medical University, Urumqi, China; 3 Department of Endocrinology, First Affiliated Hospital of Xinjiang Medical University, Urumqi, China; 4 Department of Clinical Laboratory, First Affiliated Hospital of Xinjiang Medical University, Urumqi, China; 5 Xinjiang Key Laboratory of Medical Animal Model Research, Urumqi, China

**Keywords:** Genetic linkage study, Predictive markers, Valvular disease

## Abstract

Macrophage migration inhibitory factor (MIF) is an essential mediator of atherosclerotic plaque progression and instability leading to intracoronary thrombosis, therefore contributing to coronary artery disease (CAD). In this study, we investigated the relationship between *MIF* gene polymorphism and CAD in Chinese Han population. Three single nucleotide polymorphisms (SNP, rs755622, rs1007888 and rs2096525) of *MIF* gene were genotyped by TaqMan genotyping assay in 1120 control participants and 1176 CAD patients. Coronary angiography was performed in all CAD patients and Gensini score was used to assess the severity of coronary artery lesions. The plasma levels of MIF and other inflammatory mediators were measured by ELISA. The CAD patients had a higher frequency of CC genotype and C allele of rs755622 compared with that in control subjects (CC genotype: 6.5% vs. 3.9%, *P* = 0.008, C allele: 24.0% vs. 20.6%, *P* = 0.005). The rs755622 CC genotype was associated with an increased risk of CAD (OR: 1.804, 95%CI: 1.221–2.664, *P* = 0.003). CAD patients with a variation of rs755622 CC genotype had significantly higher Gensini score compared with patients with GG or CG genotype (all *P* < 0.05). In addition, the circulating MIF level was highest in CAD patients carrying rs755622 CC genotype (40.7 ± 4.2 ng/mL) and then followed by GC (37.9 ± 3.4 ng/mL) or GG genotype (36.9 ± 3.7 ng/mL, all *P* < 0.01). Our study showed an essential relationship between the *MIF* gene rs755622 variation and CAD in Chinese Han population. Individuals who carrying *MIF* gene rs755622 CC genotype were more susceptible to CAD and had more severe coronary artery lesion. This variation also had a potential influence in circulating MIF levels.

## Introduction

Coronary artery disease (CAD), the leading cause of mortality and disability, increases the burden of healthcare costs worldwide^1^. Atherosclerosis is the major contributor of CAD characterized by chronic inflammation and lipidoses in the coronary arterial intima^2–5^. The atherosclerotic lesions often manifest as asymmetric focal thickenings of the intima, which consist of infiltrated inflammatory cells, circulating lipids, hyperplastic connective-tissue, and other kinds of debris^6^. CAD is also a multifactorial disease driven by environmental and genetic factors. Genome-wide association studies (GWAS) documented that variations in multiple genes especially inflammatory-related factors including PPARα, interleukin 18 (IL-18), IL-1β, SIRT2, and CD14 receptor were closely related to the susceptibility of CAD^7–9^.

Macrophage migration inhibitory factor (MIF), a firstly identified as T-cell cytokine, promotes atherosclerotic plaque progression and plaque instability, ultimately leading to intracoronary thrombosis^10–13^. Increased MIF expression has been observed in atherosclerotic plaque^14,15^. Pharmacological inhibition of MIF or anti-MIF antibody treatment in apolipoprotein E-deficient (ApoE^-/-^) mice markedly reduces intimal Mac-1-positive macrophages accumulation^16,17^. Moreover, MIF blockade in the ApoE^-/-^ mice stabilizes atheroma plaque manifested by decreased macrophage infiltration, foam cell formation and increased the proliferation of smooth muscle cells^16^. A similar result was also observed in low-density lipoprotein receptor-deficient (LDLR^-/-^) mice^18^. Interestingly, acute MIF release has been found as a potent cardioprotective factor in the ischemic heart via modulating activation of AMP-activated protein kinase (AMPK) pathway during a short period of ischemia^19^. However, for a prolonged ischemia (> 30 min), heightened MIF expression and production is detrimental^20^. Different cellular sources of MIF has been identified to be responsible for these distinct actions of MIF^21^. MIF levels were sharply elevated in first obtainable blood sample from ST-segment elevation myocardial infarction (STEMI) patients and the raised MIF can predict the ultimate area of myocardial infarction (MI) and the degree of adverse cardiac remodeling^22^. Elevated MIF levels were also associated with adverse long-term outcomes in patients with stable CAD and impaired glucose tolerance or type 2 diabetes mellitus^23^. Of note, different from the roles in acute myocardial injury, chronically elevated MIF exerts pro-inflammatory actions in ischemic myocardium^24^ and atherosclerosis, for example, in the development of atherosclerosis, elevated MIF promotes the recruitment of mononuclear cells and the conversion of macrophages into foam cells^18^. Together, MIF is known as a multifunctional inflammatory cytokine, playing a significant role in the progression of atherosclerosis, therefore it may be a critical contributor of CAD and acute MIF release and chronically elevated MIF play distinct roles in ischemic heart disease.

Human *MIF* gene locates in chromosome 22q11.23 and consists of three exons and two introns. Genetic epidemiology studies have demonstrated that *MIF* gene variation was associated with various inflammatory diseases including rheumatic heart disease^25^, metabolic syndrome^26^, rheumatoid arthritis^27^, ulcerative colitis^28^, psoriasis^29^, and systemic sclerosis^30^. Moreover, previous studies indicated that *MIF* gene variant was the hazardous factor for CAD among different ethnicities^10,31^, while its underlying mechanism is still unknown. In our present study, we first detected the association between the three single nucleotide polymorphisms (SNPs), rs755622, rs1007888 and rs2096525 in *MIF* gene and the CAD susceptibility in a Chinese Han population. Second, we determined the association between *MIF* gene variation and severity of CAD. Finally, we investigated potential functional consequence of *MIF* gene variant by measuring levels of inflammatory biomarkers.

## Results

### Clinical characteristics of the study population

We recruited 1176 CAD patients and 1120 controls in our study. The Clinical characteristics were shown in Table 1. We found that the CAD patients had higher levels of LDL-C, TG, TC and glucose and lower HDL-C level versus control participants (all *P* < 0.05). CAD patients were older and had a higher prevalence of hypertension and diabetes than control group (all *P* < 0.05), while gender ratio, smoking, BMI were comparable between the two groups.

**Table 1:** Clinical characteristics of including control participants and CAD patients.

Characteristics	Controls (n = 1120)	CAD patients (n = 1176)	*P* value
Age (years)	58.5 ± 11.2	59.5 ± 9.8	0.028
Men, n (%)	628 (56.1)	628 (53.4)	0.199
Smoke, n (%)	325 (29.0)	375 (31.9)	0.135
BMI (kg/m^2^)	25.7 ± 3.2	25.9 ± 3.0	0.127
Glucose (mmol/L)	5.2 ± 1.5	5.5 ± 1.5	< 0.001
TG (mmol/L)	1.5 ± 0.6	1.6 ± 0.6	0.046
TC (mmol/L)	4.4 ± 0.64	4.6 ± 0.7	< 0.001
HDL-C (mmol/L)	1.1 ± 0.2	1.0 ± 0.20	0.003
LDL-C (mmol/L)	2.4 ± 0.5	2.5 ± 0.6	< 0.001
Hypertension, n (%)	444 (39.6)	594 (50.5)	< 0.001
Diabetes, n (%)	144 (12.9)	309 (26.3)	< 0.001

Continuous variables are expressed as mean ± SD. Categorical variables are expressed as percentages. *CAD* coronary artery disease, *BMI* body mass index, *TG* triglyceride, *TC* total cholesterol, *LDL-C* Low density lipoprotein-cholesterol, *HDL-C* high-density lipoprotein-cholesterol.

### CC genotype of MIF gene rs755622 was the susceptible factor for CAD

The distributions of the three SNPs (rs755622, rs10078888 and rs2096525) in *MIF* gene were according with Hardy–Weinberg equilibrium both in CAD and control groups. The detected frequency of rs755622 CC genotype was higher in CAD patients than in controls (6.5% vs. 3.9%, *P* = 0.008). We also found that the C allele was more common in CAD patients versus controls (24.0% vs. 20.6%, *P* = 0.005, Table 2). For the other two SNPs, rs1007888 and rs2096525, we did not observe prominent difference about the distribution of genotype or allele between the two groups (all *P* > 0.05, Table 2). We further analyzed the potential risk factors for CAD by multivariate logistic regression analysis. It demonstrated that individuals with rs755622 CC genotype had a 1.8-fold higher risk of CAD than other genotypes carriers eliminating the effect of traditional risk factors including age, diabetes, hypertension, glucose and lipids (OR: 1.804, 95%CI: 1.221–2.664, *P* = 0.003, Table 3).

**Table 2:** Distribution of different genotypes and alleles of *MIF* gene variation in control and CAD participants.

Variants	Controls (n = 1120)	CAD patients (n = 1176)	*P* value
**rs755622**			
CC	44 (3.9)	77 (6.5)	0.008
CG	373 (33.3)	411 (34.9)	
GG	703 (62.8)	688 (58.5)	
C	461 (20.6)	565 (24.0)	0.005
G	1779 (79.4)	1787 (76.0)	
**rs1007888**			
TT	290 (25.9)	322 (27.4)	0.274
CT	554 (49.5)	597 (50.8)	
CC	276 (24.6)	257 (21.9)	
T	1134 (50.6)	1241 (52.8)	0.147
C	1106 (49.4)	1111 (47.2)	
**rs2096525**			
CC	45 (4.0)	42 (3.6)	0.413
CT	400 (35.7)	394 (33.5)	
TT	675 (60.3)	740 (62.9)	
C	490 (19.9)	478 (20.3)	0.728
T	1750 (80.1)	1874 (79.7)	

All the genotype frequencies were in Hardy–Weinberg equilibrium (*P* > 0.05).

The *P* value of genotype was calculated by Chi-square test. CAD, coronary artery disease.

**Table 3:** Multiple logistic regression analysis for CAD risk factors.

Factors	B	Wald	*P* value	OR	95%CI	
					Lower	Upper
rs755622 CC/GG + CG	0.590	8.796	0.003	1.804	1.221	2.664
Age	0.007	2.752	0.097	1.007	0.999	1.015
Hypertension	0.361	16.964	< 0.001	1.435	1.208	1.704
Diabetes	0.856	40.362	< 0.001	2.354	1.808	3.066
Glucose	−0.026	0.569	0.451	0.974	0.910	1.043
TG	0.026	0.067	0.743	1.026	0.879	1.199
TC	0.257	14.754	< 0.001	1.293	1.134	1.475
HDL-C	−0.438	4.389	0.036	0.645	0.428	0.972
LDL-C	0.168	4.514	0.034	1.183	1.013	1.381

*TG* triglyceride, *TC* total cholesterol, *HDL-C* high-density lipoprotein-cholesterol, *LDL-C* low density lipoprotein-cholesterol.

### CC genotype of MIF gene rs755622 was associated with severe coronary artery lesion

**Figure 1:**
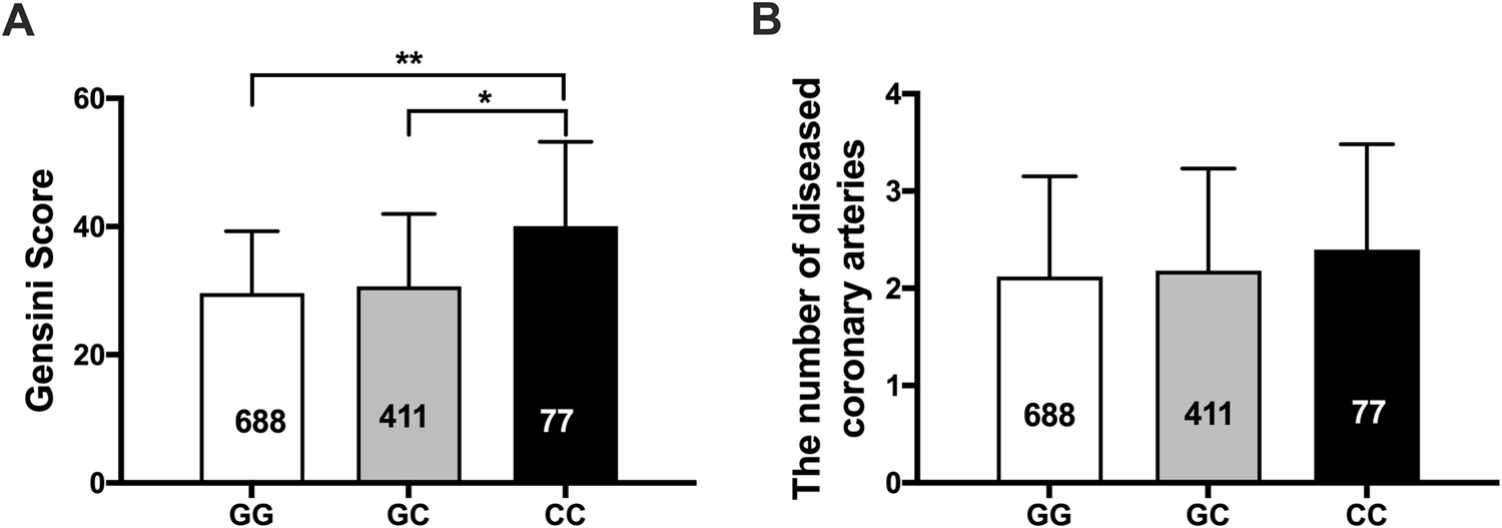
Influence of different genotypes of *MIF* gene rs755622 variation on Gensini score (A) and the number of diseased coronary arteries (B) in patients with coronary artery disease. The number in each bar indicates the group size. *P < 0.05 versus GC genotype, **P < 0.001 versus GG genotype.

We further analyzed the influence of *MIF* gene variation in coronary artery lesion in CAD patients. CAD patients with rs755622 CC genotype (40.1 ± 23.1) had a much higher Gensini score compared with patients carrying GG (29.6 ± 19.7) or CG (30.7 ± 21.3) genotype (both *P* < 0.05, Fig. 1A). After adjusting age, lipids, diabetes and hypertension by general liner model analysis, we found that Gensini score in individuals carrying rs755622 CC genotype was still higher than that in individuals carrying GG genotype (*P* = 0.001), while Gensini score was comparable between CAD patients carrying rs755622 GG and GC genotypes (*P* = 0.649, Table 4). We did not observe significant difference in Gensini score between different genotypes of rs1007888 and rs2096525 in CAD patients. For rs1007888, the Gensini score were 31.2 ± 18.4, 29.4 ± 22.1 and 33.1 ± 20.4 in patients carried CC, CT and TT genotype, respectively (*P* = 0.192). For rs2096525, the Gensini score were 28.4 ± 16.3, 29.7 ± 21.1 and 31.5 ± 20.8 in patients carried CC, CT and TT genotype, respectively (*P* = 0.479). The number of diseased arteries among the three SNPs in *MIF* gene was also comparable (Fig. 1B).

**Table 4:** General liner model analysis for Gensini score in CAD patients.

Factors	B	*P* value	t	95%CI	
				Lower	Upper
rs755622 GG	0*	–	–	–	–
rs755622 GC	0.802	0.649	0.455	−2.661	4.264
rs755622 CC	10.252	0.001	3.427	4.377	16.127
Age	0.059	0.507	0.663	−0.115	0.233
Hypertension	−2.086	0.205	−1.268	−5.316	1.144
Diabetes	1.154	0.523	0.639	−2.392	4.700
TG	0.431	0.787	0.270	−2.704	3.565
TC	−0.143	0.906	−0.119	−2.503	2.218
HDL-C	−3.555	0.373	−0.892	−11.384	4.273
LDL-C	0.614	0.681	0.412	−2.314	3.542

*This parameter was set to zero because it was redundant.

### CC genotype of *MIF* gene rs755622 was associated with elevated MIF levels

**Figure 2:**
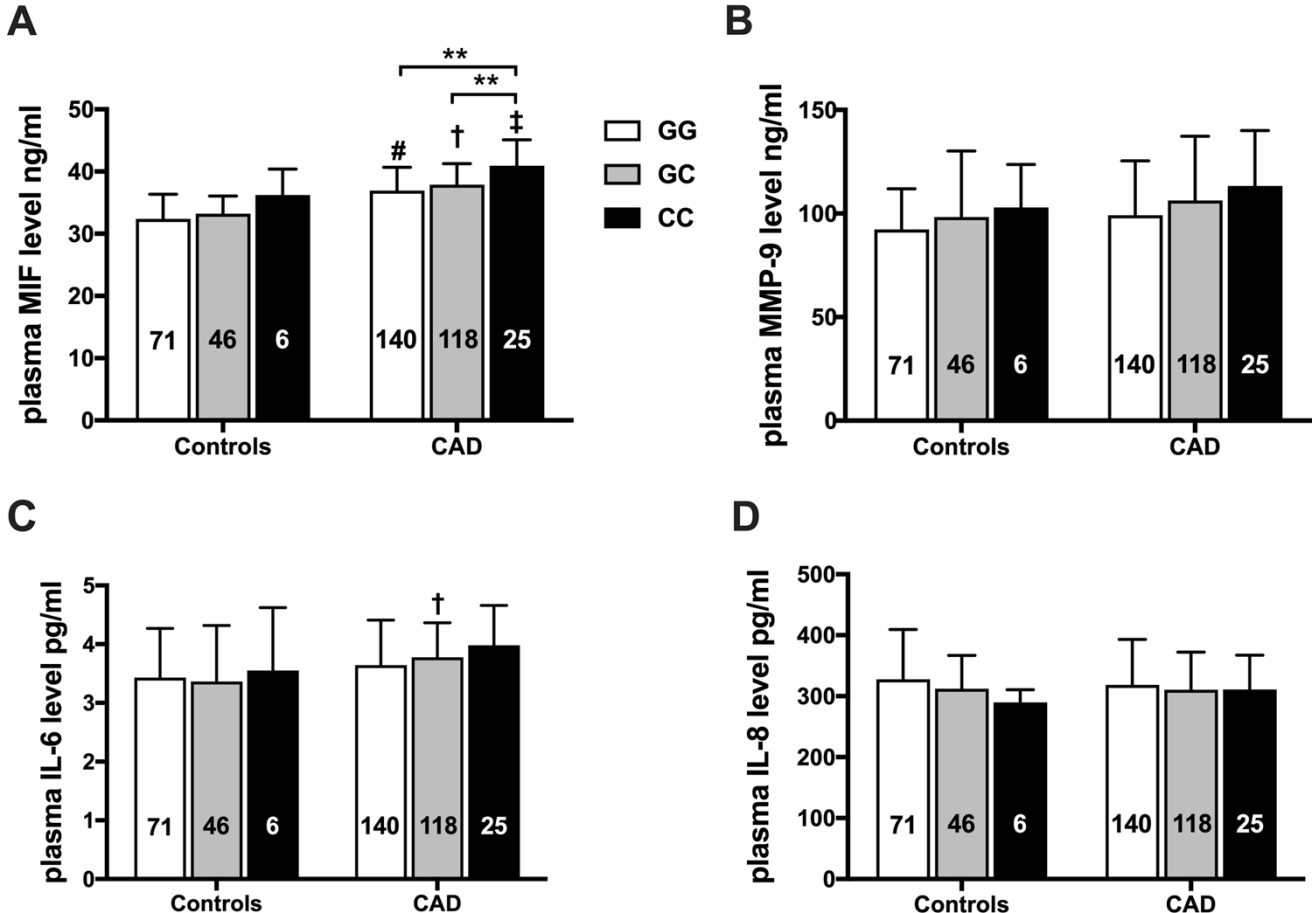
Influence of different genotypes of *MIF* gene rs755622 variation on plasma levels of MIF (A), MMP-9 (B), IL-6 (C) and IL-8 (D) in controls and patients with coronary artery disease (CAD). The number in each bar indicates the group size. *P < *0.05*, **P < *0.01*, #*P* < 0.001 versus control subjects carrying GG genotype, $\dag$*P* < 0.001 versus control subjects carrying GC genotype, $\ddag$< 0.05 versus control subjects carrying CC genotype. Two-way ANOVA was used for the statistical comparison.

We also investigated whether variation of *MIF* gene affect the expression of inflammatory cytokines such as MIF, MMP-9, IL-6 and IL-8 in randomly selected 123 controls and 283 CAD patients. The circulating MIF level in CAD patients was highest in rs755622 CC genotype carriers (40.7 ± 4.2 ng/mL) and then followed by GC (37.9 ± 3.4 ng/mL) or GG genotype carriers (36.9 ± 3.7 ng/mL, both *P* < 0.01 vs. CC genotype, Fig. 2A). These differences of MIF levels in CAD patients remained after correcting by age, lipids, hypertension and diabetes via general liner model analysis (CC vs. GG genotype *P* < 0.0001, CC vs. GC genotype *P* = 0.034). Moreover, MIF levels in CAD group were all higher than that in the counterparts of control group (all *P* < 0.05). For control participants, the circulating levels of MIF were comparable among the three genotypes of rs755622. The levels of MMP-9, IL-6 and IL-8 were comparable among the three genotypes of rs755622 in both control and CAD groups except that CAD patients carrying rs755622 GC genotype had higher IL-6 level compared with controls carrying the same genotype (*P* < 0.05, Fig. 2B-D). In addition, the levels of circulating MIF, MMP-9, IL-6 and IL-8 were comparable among CAD patients and controls carrying different genotypes of rs1007888 and rs2096525 polymorphisms (all *P* > 0.05, data were not shown).

## Discussion

CAD as a complex and multifactorial disorder is characterized by the interaction between traditionally environmental factors and genetic changes. Many genetic factors involve in the development and progression of the CAD. Recently, Schunkert and colleagues analyzed previously published GWAS and reported that 164 chromosomal loci have been identified to affect the risk of CAD. These chromosomal loci were involved in modulating traditional risk factors such as immune system and inflammation (CXCL12, HDAC9, IL5, etc.), lipid metabolism (LDLR, PCSK9, APOE, etc.), blood pressure (ARHGAP42, LPL, NOS3, etc.) and glycometabolism (IRS1, GIP, SLC22A4)^32^. Hypercholesterolemia is a primary trigger for the initiation of atherosclerosis. It leads to the accumulation of lipoproteins in the intima and also results in the formation of oxidized lipoproteins and release of inflammatory mediators^33^. Inflammation can enhance atherosclerotic plaque instability presented as plaque fissuring, erosion and rupture which provides the substrate for the thrombotic response that eventually causes myocardial ischemia or infarction^34^. The high blood pressure and elevated glucose levels are key risk factors for CAD and both can expedite the progression of atherosclerosis by impairing endothelial function^35^. MIF plays an important role in cell recruitment and arrest through binding to the chemokine receptors CXCR2 and CXCR4^36^. However, it cannot be classified into one of the four typical chemokine classes, such as CXCL12, CCL2, due to its absence of a characteristic cysteine motif in its N-terminus, and is therefore called a chemokine-like function chemokine^37^. Although the effectively clinical treatments and lifestyle modification have decreased its incidence and mortality largely, CAD was still considered the leading cause of adults mortality worldwide^38,39^. Therefore, detecting the association between susceptible gene and CAD has been considered as a valuable approach for identifying risk and improving management of CAD.

Our present study documented that the frequency of rs755622 CC genotype and C allele were more common in CAD patients than that in controls and individuals carrying CC genotype had an increased susceptibility of CAD. These findings were supported by others. It has been reported that *MIF* rs755622 C allele polymorphism was associated with CAD and the C allele might be a risk factor for CAD in other Chinese population^31,40^. The MONICA/KORA Augsburg study suggested that rs755622 C allele and rs2070766 G allele were related to the higher risk for CAD in German females after 10 years follow-up^10^. Another case–control study showed that *MIF* gene rs755622 C allele was also associated with increased risk of ACS in a Chinese Han population^41^. Our previous study which conducted in a Kazakh population, a Chinese minority ethnic, found that the frequencies of *MIF* rs755622 CC genotype and C allele were significantly higher in CAD patients than that in control subjects^42^. Some different results were also reported. Tereshchenko et al. investigated the distribution of MIF rs755622 G/C genotypes, alleles or carriage rates in Czech or Russian population, no significant difference was found between patients with MI and control subjects. However, they observed that the GG genotype of the *MIF* rs1007888 was associated with MI in Czech female patients^43^. Except the findings of rs755622 and rs1007888, another *MIF* gene SNP, rs5844572, contains a 4-nucleotide microsatellite positioned at -794 in the MIF promoter (CATT_5-8_). The CATT repeat number regulates the activity of the *MIF* gene promoter and higher CATT repeat numbers lead to stronger activity of the promoter^44^. It was reported that *MIF* − 794 CATT_5–8_ was also associated with susceptibility and severity of many in immune-related disease and CAD. A Mexican population-based case–control study documented that *MIF* -794 CATT_6/7_ genotype was associated with susceptibility to ACS^45^. Qian et al. showed that among 70 CAD patients in China, *MIF* -794 CATT_5-8_ was not associated with CAD, but carriers with -794 CATT_5_ allele showed lower plasm MIF levels than those carriers with -794 CATT_7_ allele^46^. Taken together, these findings clearly indicated the *MIF* gene rs755622 and -794 CATT_5-8_ variations were more likely to be universal genetic variants associated with increased risk of CAD.

Gensini score was a well-recognized method to evaluate the degree of coronary artery stenosis^47^. In our present study, we observed that CAD patients carrying rs755622 CC genotype had higher Gensini scores than other genotype carriers. This finding was similar to our previous study, which also indicated that CAD patients with rs755622 C allele (CC or CG genotype) had higher Gensini score compared to non C allele carriers in Kazakh population^42^. MIF is an evolutionarily ancient and highly conserved cytokine and plays as a key factor in pathogenesis of cardiovascular disease^36,48^. MIF has been identified as a major regulator of atherogenesis^49^ through the following mechanisms (1) inducing recruitment of monocytes and T cells to atherosclerotic lesions by various pro-atherogenic stimuli e.g., oxidized LDL or angiotensin II^50^; (2) regulating smooth muscle cell migration and proliferation, which may promote lesion growth and (3) increasing foam-cell transformation of macrophages and enhancing degradation of extracellular matrix proteins therefore contributing to plaque instability^51^. These results indicate that *MIF* gene rs755622 may a potential genetic marker for predicting risk and the severity of CAD.

As a pleiotropic cytokine, MIF could regulate the release of other pro-inflammatory cytokines through controls the inflammatory ‘set point’^52^. In our present study we randomly measured circulating levels of MIF, MMP-9, IL-6 and IL-8 in CAD patients and control subjects. MMP-9 is capable of degrading components of the extracellular matrix which plays an essential role in the progression and instability of atherosclerotic lesions^53^. IL-6 is a central mediator of inflammatory process^54^. Many inflammatory diseases were accompanied with elevated circulating IL-6 levels especially, in atherosclerosis, CAD, acute MI and metabolic syndromes^55^. IL-8 is also a proinflammatory cytokine that is expressed in macrophage-rich areas of atherosclerotic lesions^56^ and the elevated levels of IL-8 were associated with an increased risk of CAD^57,58^. In the present study, we observed that plasma MIF level was significantly elevated in CAD patients carrying of *MIF* gene rs755622 CC genotype. This finding was similar to our previous study, which showed MIF levels in ACS patients with CC genotype was significantly higher than ACS patients carrying GG genotype of *MIF* gene rs755622^41^. Ji et al.^40^ also reported that the plasma MIF level was higher in *MIF* rs755622 C allele carriers than in G allele carriers. Moreover, Qian et al. reported that MIF level was higher in *MIF* -794 CATT_7_ allele carriers than that of CATT_5_ allele carriers in CAD patients^46^. However, we did not observe significant difference in plasma levels of MMP-9, IL-6 and IL-8 between the 3 genotypes of rs755622. Except for CAD, an association of *MIF* rs755622 CC genotype with higher levels of MIF was also reported in other inflammatory diseases such as juvenile idiopathic arthritis^59^ and rheumatoid arthritis^27^. These results suggested that *MIF* gene rs755622 and -794 CATT_5-8_ variations had a potential impact on the expression of MIF.

There are several limitations in our study. *First*, we did not investigate the other variations of the MIF gene such as -794 CATT5-8 which had been reported positively related with CAD. *Second*, as blood samples were randomly selected, MIF and other inflammatory cytokine levels were not relevant to the severity of coronary artery lesion, therefore, we did not observe a correlation between MIF levels and Gensini score in CAD patients. *Third,* MIF levels were somewhat high in our control participants. This phenomenon may be related to other concomitant “unhealthy conditions” that might influence their baseline levels of MIF, in other words, they were not really healthy individuals. Moreover, although these control participants had no history and no signs of CAD, they might be exposed to same risk factors of CAD. *At last*, the small sample size for measuring plasma levels of MIF and other inflammatory factors limited the power of MIF genetic variation influencing expression and production of these inflammatory mediators.

In conclusion, our study demonstrated an association between the *MIF* gene variation and risk of CAD in Chinese Han population. Individuals who carried *MIF* gene rs755622 CC genotype were more susceptible to CAD and likely to have more severe coronary artery lesion. This variation also had a potential influence in circulating MIF levels.

## Materials and methods

### Study participants

Control participants were randomly selected from the Cardiovascular Risk Survey (CRS) study, which was performed in the multiethnic population in Xinjiang, northwestern part of China from October 2007 to March 2010^60,61^. Individuals with no history of cardiovascular disease and no signs of CAD according to their physical and laboratory examination, electrocardiograph and echocardiography were defined as control participants. But these control participants were not the real healthy individuals. They may have hypertension, dyslipidemia and diabetes, which means these controls exposed to same risk factors of CAD. This study recruited CAD patient during May 2009 and December 2014. CAD patients were recruited from inpatients who received treatment in the First Affiliated Hospital of Xinjiang Medical University. CAD was considered as the presence of coronary artery stenosis (> 50% lumen narrowing) in one or more major coronary arteries according to coronary angiography^42^. Angiographic evaluations were reviewed by two independent interventional cardiologists blinded to the study information. Gensini score was applied to evaluate the severity of CAD as we described previously^42^. We also counted the number of diseased arteries. Patients with more than one major coronary artery stenosis were defined as multi-vessel disease. Exclusion criteria for both CAD and control participants were those with concomitant valvular heart disease, congenital heart disease, non-ischemic cardiomyopathy and acute and chronic inflammatory diseases. As valvular heart disease, congenital heart disease and non-ischemic cardiomyopathy were also involved in genetic disorders, which may be confounding factors interfering with the association between *MIF* gene variation and susceptibility of CAD.

### Definition of cardiovascular risk factors

Body mass index (BMI) was calculated by dividing body weight (in kilograms) with the height in meters squared. Individuals who smoked regularly in the past six months were regarded as current tobacco users. A person whose systolic blood pressure exceeded 140 mmHg and/or diastolic blood pressure was more than 90 mmHg that occurred twice in different occasions was defined as a hypertension^62^. Diabetes was defined if a person has history or presence of diabetes with blood sugar lowering medicine in use or a fasting plasma glucose > 7.0 mmol/L (126 mg/dL) on 2 separate occasions or a random glucose value of > 11.1 mmol/L (200 mg/dL) according to the World Health Organization criteria^63^.

### Laboratory Determinations

After overnight fasting (at least 8 h), 5 mL peripheral venous blood were drawn from all participants in the morning around 8–10 am. Blood cells and plasma were separated by centrifugation and then they were stored at -80℃ for genotyping and biochemical assay. Biochemical measurements included blood glucose and lipids including total cholesterol (TC), low density lipoprotein-cholesterol (LDL-C), high density lipoprotein- cholesterol (HDL-C), and triglycerides (TG). All of the plasma samples were measured by standard enzymatic methods in the Central Laboratory of the First Affiliated Hospital of Xinjiang Medical University.

### Enzyme linked immunosorbent assay (ELISA)

We measured plasma levels of MIF, IL-6, IL-8, and matrix metalloproteinase-9 (MMP-9) in randomly selected control subjects and CAD patients to investigate the potential functional influence of *MIF* gene variation by ELISA kits (Wuhan USCN Business Co., Ltd, China). The randomization of participant selection for ELISA was performed by following the computer generated sequential numbers (blocked randomization) and the participants’ allocations were kept in sealed envelopes according to our previously established method^64^.

### DNA extraction and SNPs selection

We utilized a commercial whole blood genome extraction kit (BioTeke Corporation, Beijing, China) to extract DNA from peripheral blood leukocytes according to the manufacturer’s instructions. Human MIF gene contains 840 bp and locates in the chromosome 22 (chr22:23,894,383–23,895,223). We utilized Haploview 4.2 software to select the TagSNPs of *MIF* gene based on Hapmap human SNP database (CHB population data). We got 50 SNPs in *MIF* gene which their minor allele frequency ≥ 0.1. We captured 8 TagSNPs by setting linkage disequilibrium patterns with r^2^ ≥ 0.8, which including rs755622, rs1007888 and rs2096525^65^. rs755622, also known as -173G/C polymorphism, locates in the promoter region (chr22:24,236,392), rs1007888 (chr22:24,241,101) locates in the translation termination codon and rs2096525 (chr22:24,236,819) locates in the first intron.

### Genotyping

As presented in our previous study^42^, we used TaqMan SNP genotyping assay (Applied Biosystems) to genotype the polymorphism of *MIF* gene. The primers and probes used in the assay were chosen according to the information at the ABI website (http://myscience.appliedbiosystems.com). We chose Applied Biosystems 7900HT Standard Real-Time PCR System for the DNA amplification and used Sequence Detection Systems automation controller software v2.3 (ABI) to genotype these three SNPs. The reaction system of PCR amplification was as follows: 3 μL of TaqMan Universal Master Mix, 0.12 μL probes and 1.88 μL ddH_2_O in a 6 μL final reaction volume containing 1 μL DNA (50 ng). Amplification cycling conditions were as follows: 95 °C for 5 min; 40 cycles of 95 °C for 15 s; and 60 °C for 1 min.

### Statistical analysis

Analyses were carried out by SPSS version 17.0 (SPSS Inc., Chicago, IL). Continuous variables were presented as mean ± standard deviation (SD) and the differences between CAD and controls was detected by Student unpaired *t* test between. Categorical variables were showed as proportions and their differences between the two groups were analyzed by chi-square test. Chi-square test was also used in the analysis of Hardy–Weinberg equilibrium. The association between *MIF* gene variation and CAD was detected by multiple logistic regression analysis. The difference in the levels of MIF, MMP-9, IL-6 and IL-8 and Gensini score was analyzed by two-way ANOVA among different genotypes. General linear model analysis was performed to test the associations among different genotypes, plasma levels and Gensini score. *P* < 0.05 was considered as statistical significance.

### Ethical approval

Written informed consent was obtained from all of study participants. This study was approved by the Ethics Committee of the First Affiliated Hospital of Xinjiang Medical University (Ethic Approval No. 20070726–8) and conducted according to the standards of the Declaration of Helsinki.
